# Assessment of resectability of pancreatic cancer using novel immersive high-performance virtual reality rendering of abdominal computed tomography and magnetic resonance imaging

**DOI:** 10.1007/s11548-023-03048-0

**Published:** 2024-01-22

**Authors:** Julia Madlaina Kunz, Peter Maloca, Andreas Allemann, David Fasler, Savas Soysal, Silvio Däster, Marko Kraljević, Gulbahar Syeda, Benjamin Weixler, Christian Nebiker, Vincent Ochs, Raoul Droeser, Harriet Louise Walker, Martin Bolli, Beat Müller, Philippe Cattin, Sebastian Manuel Staubli

**Affiliations:** 1 Faculty of Medicine, University of Basel, Klingelbergstrasse 61, 4056 Basel, Switzerland; 2 Institute of Molecular and Clinical Ophthalmology Basel (IOB), Mittlere Strasse 91, 4031 Basel, Switzerland; 3 Clarunis, University Center for Gastrointestinal and Liver Diseases, 4002 Basel, Switzerland; 4 Department of Radiology St. Claraspital Basel, Kleinriehenstrasse 30, 4058 Basel, Switzerland; 5 Department of HPB Surgery and Liver Transplantation, Royal Free Hospital, London, NHS Foundation Trust, Pond Street, London, NW3 2Q UK; 6 Department of General, Visceral and Vascular Sugery, Charité Campus Benjamin Franklin, Hindenburgdamm 20, 12203 Berlin, Germany; 7 Surgical Department, Cantonal Hospital Aarau, Tellstrasse 25, 5001 Aarau, Switzerland; 8 Department of Biomedical Engineering, University of Basel, Hegenheimermattweg 167c, 4123 Allschwil, Switzerland; 9 Department of Ophthalmology, University of Basel, 4031 Basel, Switzerland; 10 Moorfields Eye Hospital, NHS Foundation Trust, London, EC1V 2PD UK; 11 Department of Women’s Health, University College London Hospitals, London, UK

**Keywords:** Virtual reality, Anatomy, Innovation, Medical training, Pancreatic cancer

## Abstract

**Purpose:**

Virtual reality (VR) allows for an immersive and interactive analysis of imaging data such as computed tomography (CT) and magnetic resonance imaging (MRI). The aim of this study is to assess the comprehensibility of VR anatomy and its value in assessing resectability of pancreatic ductal adenocarcinoma (PDAC).

**Methods:**

This study assesses exposure to VR anatomy and evaluates the potential role of VR in assessing resectability of PDAC. Firstly, volumetric abdominal CT and MRI data were displayed in an immersive VR environment. Volunteering physicians were asked to identify anatomical landmarks in VR. In the second stage, experienced clinicians were asked to identify vascular involvement in a total of 12 CT and MRI scans displaying PDAC (2 resectable, 2 borderline resectable, and 2 locally advanced tumours per modality). Results were compared to 2D standard PACS viewing.

**Results:**

In VR visualisation of CT and MRI, the abdominal anatomical landmarks were recognised by all participants except the pancreas (30/34) in VR CT and the splenic (31/34) and common hepatic artery (18/34) in VR MRI, respectively. In VR CT, resectable, borderline resectable, and locally advanced PDAC were correctly identified in 22/24, 20/24 and 19/24 scans, respectively. Whereas, in VR MRI, resectable, borderline resectable, and locally advanced PDAC were correctly identified in 19/24, 19/24 and 21/24 scans, respectively. Interobserver agreement as measured by Fleiss κ was 0.7 for CT and 0.4 for MRI, respectively (*p* < 0.001). Scans were significantly assessed more accurately in VR CT than standard 2D PACS CT, with a median of 5.5 (IQR 4.75–6) and a median of 3 (IQR 2–3) correctly assessed out of 6 scans (*p* < 0.001).

**Conclusion:**

VR enhanced visualisation of abdominal CT and MRI scan data provides intuitive handling and understanding of anatomy and might allow for more accurate staging of PDAC and could thus become a valuable adjunct in PDAC resectability assessment in the future.

**Supplementary Information:**

The online version contains supplementary material available at 10.1007/s11548-023-03048-0.

## Introduction

**Fig. 1:**
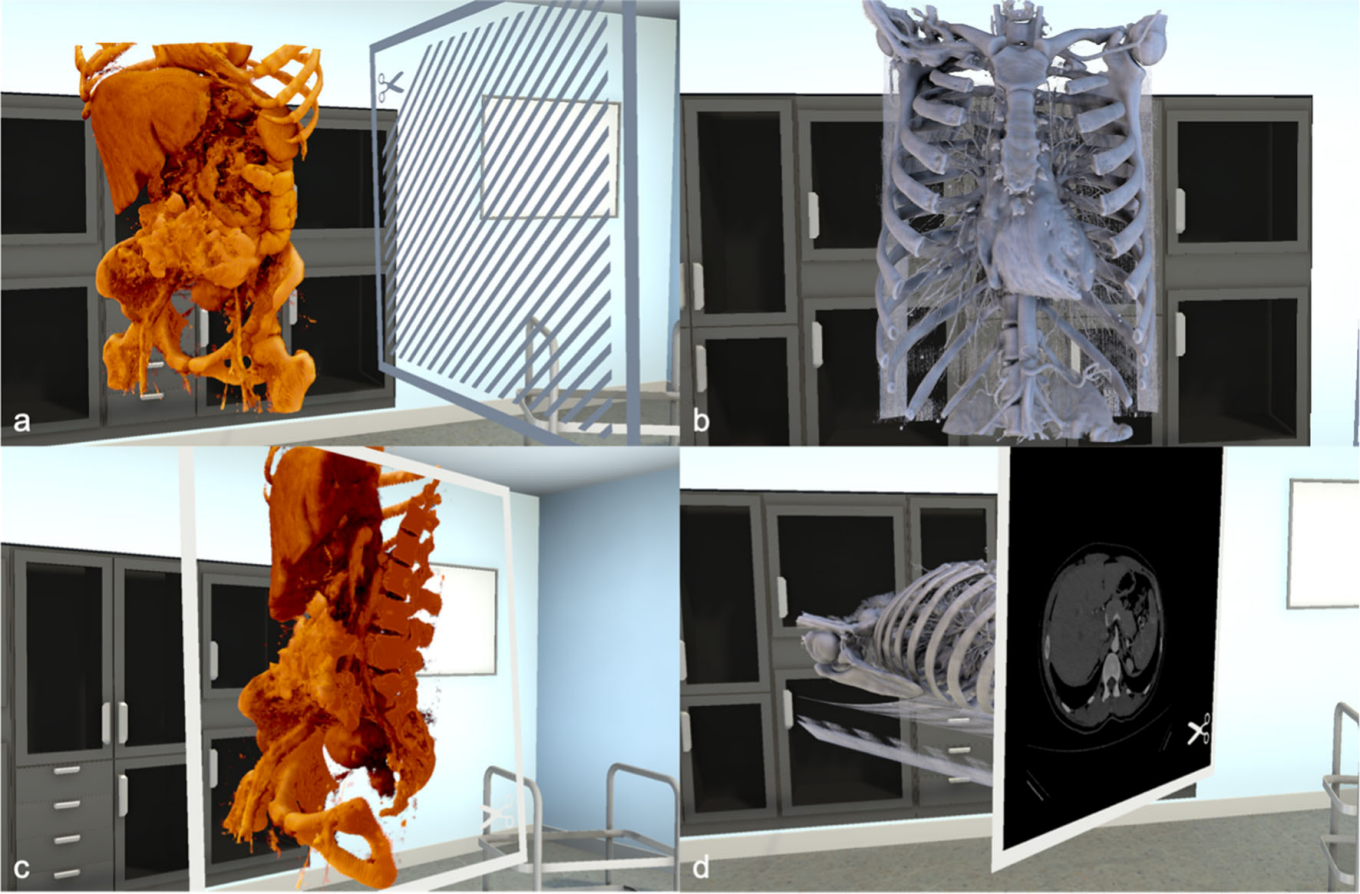
Visualisation of the basic display options **a**, **b** Images from different modalities can be loaded into the VR environment. The models can then be placed, moved and rotated as desired. **c** Sectioning of the 3D model is possible using the shown cutting plane **d** The cutting plane can be switched, so that the matching section of the respective modality is shown

Virtual reality (VR) allows the viewer to experience a computer-generated environment interactively [1, 2]. Recently, a picture archiving and communication system (PACS) compatible VR software has been developed (Specto VR™), capable of rendering, segmenting, and displaying cross-sectional medical imaging such as Computed Tomography (CT) or Magnetic Resonance Imaging (MRI) in real-time [3]. In essence, this software can be used to import a cross-sectional imaging dataset directly from PACS and render this image data into a freely interactive 3D model, allowing detailed study of the data in a VR environment. The original dataset can be superimposed simultaneously with the rendered volume model by the usage of a cutting plane. (Fig. 1) This software has previously been validated in various settings, including ophthalmological imaging as well as Magnetic Resonance Cholangio-Pancreatography (MRCP) [4, 5]. In these studies, the VR application was shown to be safe, was well tolerated, improved the anatomical understanding, and even potentially introduced a clinical benefit when used as a preoperative tool for trainee surgeons.

Even though VR visualisation is currently explored extensively, translation into daily clinical practice on a wide scale has not yet occurred [6, 7]. In the future, it is likely that VR enhanced visualisation of abdominal CT and MRI data will gain further importance [8]. In the past, the usage of VR anatomical models to support learning and understanding of anatomy has been shown to be beneficial [9]. However, recognising anatomical structures in VR CT and MRI imaging of real patients is a necessary cornerstone to facilitate the clinical implementation of this technology. In the future, application of VR technology to assess complex medical images such as vascular involvement of pancreatic ductal adenocarcinoma (PDAC) could improve surgical and oncological treatment planning. In PDAC, the surgical resectability decision is mainly based on two-dimensional cross-sectional image assessment, which is a challenge in clinical practice due to notoriously low inter-observer agreement (7.2%-30% for CT scans, Fleiss κ range 0.282–0.555) [10, 11]. According to the current National Comprehensive Cancer Network (NCCN) guidelines, three categories of resectability exist: Resectable (R), borderline resectable (BR) and locally advanced (LA) disease [12]. (Table 1).

**Table 1:** Definition of resectability as per NCCN Guidelines for pancreatic head cancer

	Localised and resectable (R)	Borderline resectable (BR)	Locally advanced and not resectable (LA)
A	Clear fat planes around CA, HA, SMA	Encasement of GA up to HA with either short segment encasement or direct abutment of HA without extension to CA, tumour abutment of the SMA which does not exceed greater than 180° of the circumference of the vessel wall	Aortic invasion or encasement of more than 180° of SMA, any celiac axis or IVC abutment, encasement of the SMA or CA
V	No distortion of SMV / PV	Venous involvement of the SMV / PV with distortion or narrowing or occlusion of the vein with suitable vessel proximal and distal, allowing for safe resection and replacement	Not reconstructible SMV/PV

*A* arterial, *V* venous, *CA* coeliac artery, *HA* hepatic artery, *SMA* superior mesenteric artery, *GA* gastroduodenal artery, *IVC* inferior vena cava, *SMV* superior mesenteric vein, *PV* portal vein, *NCCN* National Comprehensive Cancer Network

Of note, the resectability of PDAC likely represents a clinical continuum rather than a clear cut-off, further adding to the complexity or resectability assessment. Nonetheless, current guidelines recommend contrast-enhanced CT or MR imaging to assess local resectability, and VR software might add potential benefit by allowing the viewer to freely interact with 3D rendered cross-sectional imaging as well as viewing the original dataset.

In this study, we aim firstly to assess the general capability of clinicians from various backgrounds, both in terms of clinical experience as well specialisation, to understand the displayed anatomy with immersive, 3D-rendered VR CT and MRI visualisation. In the second step, this technology is tested for its usability as a tool to assess vascular involvement and ultimately, the resectability of PDAC by expert abdominal surgeons and radiologists.

## Methods

This prospective study was conducted according to the Declaration of Helsinki in Basel, Switzerland at Clarunis (University Centre for Gastrointestinal and Liver Disease), consisting of the abdominal surgery units of St. Claraspital (SCS) and the University Hospital in Basel as well as the Royal Free Hospital, London, UK. The Ethics Committee of Northwestern and Central Switzerland approved the use of patient data (Ethikkommission Nordwest und Zentralschweiz, EKNZ 2021-00457; AO_2021-00053). The study participants who were medical professionals provided written informed consent. Patients contributing data either signed a written informed consent form issued by the EKNZ or data was used if they had previously signed the institutions general research consent.

### Participants

In the first study step, all clinicians from the named institution were eligible to participate. In the second study step, abdominal surgeons and radiologists who had completed their training or were undergoing specialist Hepatobiliary and Pancreatic surgical subspecialisation were eligible for study participation. Study participants were recruited via a written invitation to the surgical, medical, radiological, and gastroenterological departments or via personal invitation. Study participation was voluntary without financial compensation. Study procedures were explained verbally, and participants gave written and oral informed consent prior to study inclusion. Each participant was assigned a participation number. The literature on usability testing determined the number of study participants [13].

### Study procedures

The study was divided into two steps. In the first study step, a pre-test survey to obtain demographical data, professional experience and previous exposure to VR was completed by the participants. Each study participant was individually exposed to the VR experiments. After fitting of a head-mounted display (HMD) to the participants’ head, they were allowed to acclimatise to the VR environment. Detailed instructions of the usage of the system were given. Then, a 3D VR CT model was displayed. To measure correct detection of requested anatomical structures, participants were asked to highlight specific anatomical structures with a cursor built into the VR system that could be observed on the computer screen, and the correctness of the answer was recorded. The same procedure was repeated for the 3D VR MRI model. The study personnel monitored all actions of the participants in the VR surroundings on an additional screen. A short post-test survey was handed out upon completion of the VR anatomy identification task with two open questions were asked: (1) What did you like most? (2) What did you like least?. No limitation to the length of the answers was given.

In the second study step, imaging data from 34 patients with a pathology or cytology proven PDAC and MRI or CT staging were selected from the internal hospital cancer database, with a comparable number of R, BR, and LA cases on imaging as defined by the NCCN [12].

Clinical reports, preoperative imaging, postoperative outcomes as well as multidisciplinary team (MDT) outcomes were collected for all patients included in the study. MDT discussions were chaired by the clinical leads or their deputies of the surgical, oncological, radiological, and radiooncological departments, who had to reach consensus regarding resectability and treatment of the patients. Mean work-experience post-completion of clinical training of the senior members of each units was 22 years. Abdominal surgeons and radiologists were recruited for demonstration of a set of 6 individually randomly selected VR CT and 6 VR MRI scans. Each participant assessed 2 scans with R, BR and LA PDAC per imaging modality. The participants were allowed free usage of all the VR software’s features, including the cutting plane and no time limit was given. Time measured from beginning of image display to final verdict of vascular involvement and resectability status was recorded by the study personnel. Two months after the conduction of these experiments, study participants were shown the same CT scans in standard 2D PACS, serving as control group. Correct assessment of vascular involvement (R, BR, LA) as well as time to answer were measured. The candidates’ answers were compared to the official imaging reports from the respective radiology departments as well as the MDT decision reports.

### VR software and equipment and radiological imaging

The VR application used (Specto VR™, Version 4.0, Specto Medical, Basel, Switzerland) allows imaging data to be imported and displayed in real-time, enhanced by real-time ray casting. Specto VR™ displays volume rendered images at 90 frames per second per eye. Colour transfer functions can be freely be adjusted to visualise different tissues. A cutting plane with free adjustability was provided to display the original dataset (cross-sectional slices) on demand, and to interact with the original dataset. Free rotation in every direction of the VR model as well as zooming in or out is possible. (Fig. 1) To run this application, an ASUS ROG Zephyrus GX501GI-EI005T (15.60″; full HD; Intel Core i7-8750H, 16 GB; 512 GB hard-drive; and graphics processing unit Nvidia GTX1080 MaxQ) laptop computer was used. Two VR HMD were used (HTC Vive, Xindian District, New Taipei City, Taiwan) and HP Mixed Reality (Hewlett-Packard, Palo Alto, California, USA).

In the used cross-sectional imaging, individual voxel size was: X: 0.799 (mean) ± 0.178, Y: 0.799 (mean) ± 0.178, Z (slice thickness): 2.433 ± 0.751. The Interslice gap was −0.461 (mean) ± 0.560. In case of overlapping slice reconstruction, negative interslice gap values are indicated.

Used MRI models were rendered T1 images with fat suppression using radio-frequency–spoiled 3D GRE sequences. For CT, the used sequences were late arterial or portal venous and as arterial, depending on the protocol used in the respective radiological department. Study participants were allowed to freely study the arterial as well as portal venous or late arterial phases, depending on availability. Appendix 1.

### Statistical analysis

All variables are expressed as the median and interquartile range, or counts (percentages), unless otherwise specified. The descriptive nature of the data was confirmed by an independent statistician. Microsoft Excel™ v16.68 (Microsoft, Redmond, Washington, USA) was used for descriptive analysis. Correlation analysis was performed using the Spearman’s rank correlation for paired and independent variables. Inter-rater agreement analysis was conducted with Fleiss Kappa. Differences among proportions derived from categorical data were compared using the Pearson Chi Square (*χ*^2^) test. Two-group comparison of normally distributed data was performed by the Student’s *t* test. Comparison between small sample sizes and non-normal distributed data was performed with the Mann–Whitney U test. Statistical analysis was performed with R version 3.3.2 (The R Project for Statistical Computing, GNU General Public License version 2) and R Studio version 1.0.44 (RStudio) with the graphical user interface, rBiostatistics.com [14].

## Results

### Subjects

In the first study step, the majority of subjects were abdominal surgeons (31/34), 3 were radiologists, specialised in abdominal imaging (3/34). Median age was 37 years (IQR 37–45) and fourteen were female (14/34). Median work experience was 11 years (IQR 0.33–17). The majority had finished their training (24/34). A minority had previous experience with VR (9/34), the rest of the participants was VR-naïve (25/34).

In the second study step, 9 subjects were abdominal surgeons and 3 were radiologists. One was female, and median age was 40 years (IQR 36–46). Median work experience was 14 years (IQR 9–20) and all participants had completed their training. In the 2D PACS control group, a total of 5 surgeons participated with a median age of 39 (IQR 34–41) and a median work experience of 13 years (IQR 6–15). Control group and study group did not differ significantly in age (*p* = 0.927) and work experience (p = 0.783).

### Recognisability of abdominal anatomy in VR CT and VR MRI

In VR CT, all 34 participants were able to detect all the tested solid organs (liver, spleen, kidneys) with a lower rate for the pancreas (30/34). All vascular structures were correctly identified by all of the participants (aorta, inferior vena cava, portal vein, coeliac trunk, portal vein, splenic artery, and common hepatic as well as the superior mesenteric artery).

In VR MRI, all the participants were able to detect all of the queried solid organs. All the participants correctly identified the vascular structures listed apart from the common hepatic artery (18/34). (Table 2).

**Table 2:** Results of correctly identified anatomical structures

Assessed structure	VR CT (%)	VR MRCP (%)
Pancreas	30 (88)	34 (100)
Liver	34 (100)	34 (100)
Spleen	34 (100)	34 (100)
Kidneys	34 (100)	34 (100)
Aorta	34 (100)	34 (100)
Inferior vena cava	34 (100)	34 (100)
Portal vein	34 (100)	34 (100)
Coeliac trunk	34 (100)	34 (100)
Splenic artery	34 (100)	31 (91)
Common hepatic artery	34 (100)	18 (53)
Superior mesenteric artery	34 (100)	34 (100)

Percentage of positive responses in brackets

### Vascular involvement of PDAC and assessment of resectability in VR

All twelve participants reached a verdict for each scan regarding vascular tumour contact, except in one case. A lack of response was recorded as an erroneous answer. A correct response was defined as consistency with the written MDT assessment. Median time for the assessment of VR CT and MRI scans was 185 s (IQR 138–212) and 116 s (IQR 95–152), respectively. Median assessment time for 2D PACS CT was 110 s (IQR 60–154). Median correct answers per participant (maximum 6 per imaging modality) in VR CT and MRI were 5.5 (IQR 4.75–6) and 5 (IQR 4.75–5), respectively. In 2D PACS CT viewing, median correct answers per participants were 3 (IQR 2–3). Compared to the 3D VR group, significantly fewer correct answers were given in the 2D PACS CT group whilst interacting a significantly shorter amount of time (*p* < 0.001).

In VR CT, R, BR, and LA PDAC was identified in 22/24 (92%), 20/24 (83%) and 19/24 (79%) scans, respectively. In VR MRI, R, BR, and LA PDAC was identified in 19/24 (79%), 19/24 (79%) and 21/24 (88%) scans, respectively. In 2D PACS CT, R, BR and LA PDAC was identified in 6/10 (60%), 2/10 (20%) and 4/10 (40%) scans, respectively. Five out of six participants preferred VR CT over VR MRI for the assessment of resectability. Correlation analysis between work experience and number of correct answers did not reveal a correlation with *ρ* = −0.18 (*p* = 0.41). Interobserver agreement as measured with Fleiss κ was 0.7 (*p* < 0.001) for VR CT, indicating substantial agreement. For VR MRI, κ was 0.4 (*p* < 0.001), indicating fair agreement. For 2D PACS CT, κ was 0.04 (*p* = 0.48), indicating slight agreement.

**Fig. 2:**
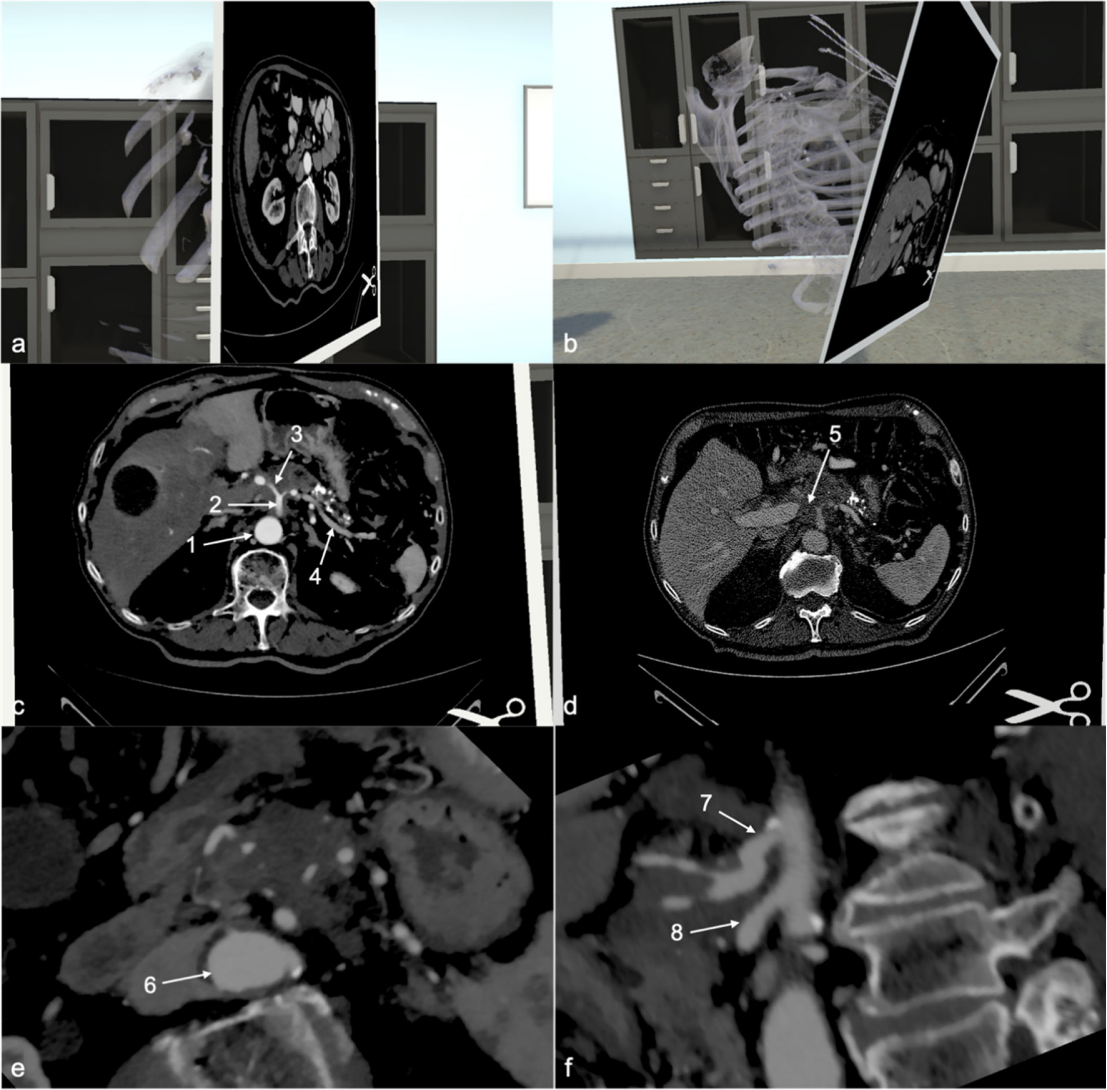
CT model of locally advanced pancreatic cancer. **a** Section through the CT model of a patient with LA pancreatic cancer. **b** The cutting plane can be placed in any desired angle. **c** Overview of an axial section: (1) Aorta (2) Celiac trunk (3) Common hepatic artery (4) Splenic artery. **d** Light–Dark contrast can be adapted for each model. The tumour is clearly visible on this section (5). **e**, **f** Tumour encasement of vessels is seen in various sections from different angles: (6) Aorta (7) Celiac trunk (8) Superior mesenteric artery

**Fig. 3:**
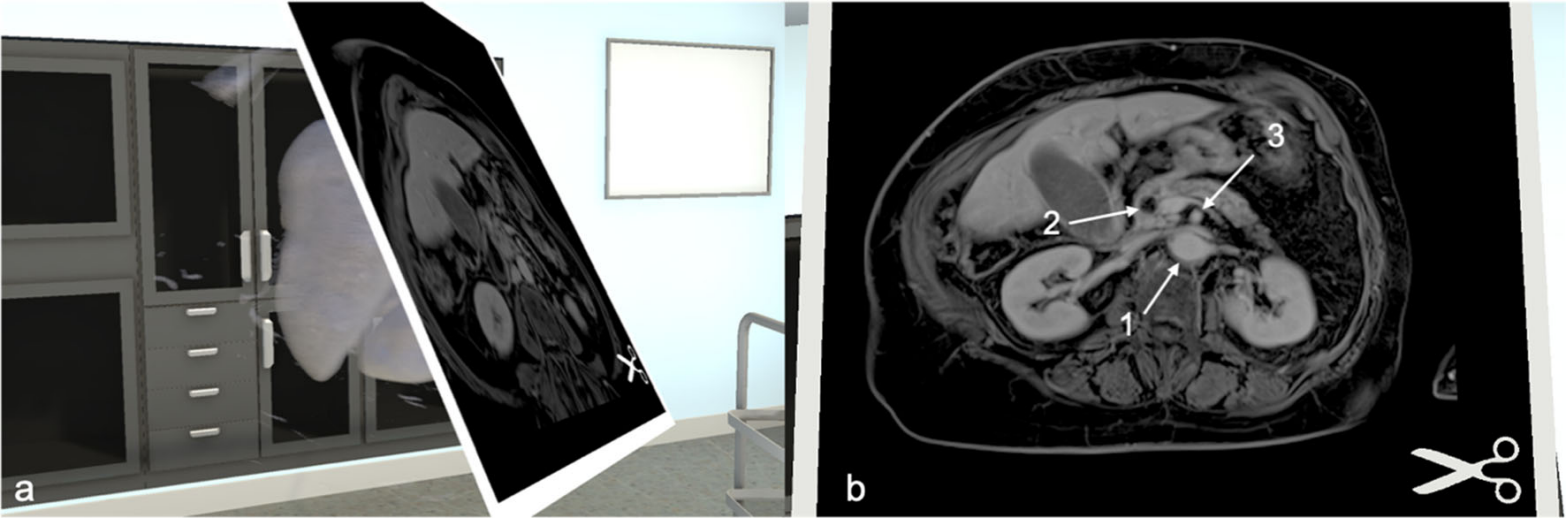
MR model of resectable pancreatic cancer. Similar to the CT model, the same assessment can be made for an MR model. **a** Section through the MR model of a patient with resectable pancreatic cancer. **b** Example of an axial section without visible tumour mass: (1) Aorta (2) Biliary duct (3) Superior mesenteric artery

The details of the differing interpretations of scans are discussed in Table 3 (Fig. 2, 3).

**Table 3:** Results from VR CT and MRI with lack of interobserver agreement (participant vs. MDT/radiology report)

Modality	Group	Radiology report	Interobserver disagreement (VR vs. MDT/radiology report)	Clinical remarks
CT	LA	Large tumour originating from pancreatic body, infiltrating stomach, duodenum, encasing coeliac trunk	Tumour not identified by 2 participants	Palliative chemotherapy
CT	BR	Solid contact of tumour to SMA (< 90°) and SMV	Deemed resectable by participant, vascular contact not identified	Patient underwent Whipple’s procedure, R0 resection, no vascular involvement intraoperatively
CT	LA	Large HOP mass, infiltrating mesenteric root, encasing SMA, infiltration of PV	Tumour not identified by participant	Palliative chemotherapy
MRI	BR	HOP mass, infiltrating SMV and solid tumour contact with SMA (< 180°)	Deemed resectable by participant	Neoadjuvant treatment, laparotomy, exploration, procedure abandoned due to tumour infiltration over long section of SMA
MRI	R	2.6 HOP mass, no visible infiltration of surrounding vascular structures	Participant unsure if tumour contact to SMV/PV present	Whipple’s procedure performed, R0 resection, no vascular infiltration intraoperatively
MR	LA	5 cm HOP mass with 180° contact to CHA, possible contact to celiac trunk and occlusion of SMV/PV confluence	Participant judges situation as borderline resectable	Palliative chemotherapy
MR	BR	4.7 cm HOP mass with contact to SMV. Previous right hemicolectomy noted with central lymphadenectomy and removal of inferior mesenteric vein, presence of clip noted. Scarring and imaging artefacts in the area	Participant can’t clearly delineate tumour or assess contact to vasculature	Whipple’s procedure performed, R0 resection, tumour lifted off SMV with minimal adherence, ileotransversostomy adherent to pancreatic head, resected, end ileostomy formed. Adjuvant chemotherapy. Early recurrence with distant metastases (pulmonary)
MR	R	2.4 cm HOP mass, < 90° contact of HOP to SMV, other vessels without tumour contact	Participant can’t give a final answer on vascular involvement	Whipple’s procedure performed. R0 resection

*R* resectable, *BR* borderline resectable, *LA* locally advanced, *CT* computed tomography, *MRI* magnetic resonance imaging, *HOP* head of pancreas, *SMA* superior mesenteric artery, *CHA* common hepatic artery, *PV* portal vein, *SMV* superior mesenteric vein, *MDT* multidisciplinary team

### Participant feedback

Participant feedback in the form of a post-survey was collected in free text form. 31 out of 34 participants provided an answer to the question “what did you like the most?” and 20 out of 34 participants provided an answer to the question “what did you like the least?”. Mainly, participants enjoyed the ease of handling (10/34), quality of 3D imaging (10/34), improved understanding of anatomy (7/34) and the free movability of the reconstructed model (5/34). In contrast, the image resolution (9/34) and weight of the headset (8/34) were the main criticisms issued by the participants. (Table 4).

**Table 4:** Summarised results of the free text feedback form

What did you like most?	What did you like least?
Ease of handling / intuitive handling (10)	Resolution (9)
Quality of 3D imaging (10)	Headset heavy (8)
Improved understanding of Anatomy (7)	Not all structures in an anatomic region (1)
Free movement (5)	Can't see what happens around me (1)
Novelty of experience (4)	Vertigo and dizziness (1)
Spatial representation of anatomy (3)	
Cutting plane function (3)	
Realistic experience (3)	
Experience overall (2)	
Useful for surgical planning (2)	
Performance of software (1)	

Numbers of times answer given in brackets

In the second study step, the majority of the participants stated that the anatomy displayed was easy to understand (11/12), that VR can improve patient treatment (12/12), can help to anticipate intraoperative difficulties (9/12), and represented overall an enjoyable experience (12/12). No significant correlation between positive answers and performance as measured by correct answers was found for above named items with *ρ* = 0.05 (*p* = 0.88), *ρ* = 0.12 (*p* = 0.69), *ρ* = −0.21 (*p* = 0.52), and *ρ* = −0.12 (*p* = 0.7), respectively.

## Discussion

In this pilot study, 3D VR-enhanced abdominal CT and MRI were able to display the anatomy in an understandable way, both for junior and experienced medical professionals. The VR experience was received positively by the tested study population. Experienced surgeons and radiologists were able to assess the vascular involvement and, ultimately, the resectability of PDAC in the majority of cases presented. Direct comparison between 3D VR enhanced and standard 2D PACS viewing of CT imaging displaying PDAC showed significantly higher accuracy in the VR group.

The first part of the study aimed at assessing the understandability of 3D VR enhanced cross-sectional abdominal imaging. There are only a few studies that directly assess the comprehensibility of VR-enhanced abdominal CT or MRI anatomy [15, 16]. Previously, a VR-enhanced CT scan was reported in a feasibility study, however, the software automated segmentation and PACS import was not available. For the implementation of VR as a clinical adjunct, the accuracy and comprehensibility of VR-enhanced visualisation will need to be further scrutinised to guarantee patient safety. This study aims to lay a cornerstone for this purpose, as most participants, including junior doctors could reliably identify the displayed anatomy. In the past, this VR software has been evaluated as a tool to teach anatomy, indicating that anatomical learning was perceived as more efficient and engaging when compared to the standard anatomy learning with models or books [17]. A recently published meta-analysis including a total of 15 randomised trials that evaluated the efficacy of VR-supported anatomy teaching, concluded that post-VR intervention anatomical test scores were significantly higher when compared with other teaching methods [9]. In these studies, anatomical VR models were used, but not PACS imported imaging datasets. Overall, these results align with previously published results from our group, showing that VR MRCP led to faster and more accurate anatomical understanding when compared to printed MRCP scans [5].

Although the participant’s feedback was largely positive, and more participants provided positive than negative feedback, a few criticisms must be addressed. The main criticism of the VR experience was a perceived lack of image resolution. The used VR software imports isovoxels from the original dataset in a 1:1 fashion, thus the resolution is limited not by the VR software, but by the original dataset. In VR, the model can be zoomed in and magnified to an extent where the VR model becomes larger than the scanned person in real life. By comparison, most screens used are relatively small, leading to the viewers perception of sufficient image resolution as compared to the magnification possible in the VR system. In the future, artificial intelligence might be introduced to improve appearances and resolution of imaging, however, this may come with new difficulties and problems as the original dataset could be altered [18]. Another comment issued by the participants was the weight of the headset. Although a recognised problem, recent advances in hardware development will likely render this issue obsolete and should not be seen as a deterrent for future use of VR in a clinical setting [19].

In the second part of the study, this VR software was evaluated as a tool to assess the presence or absence and extent of vascular contact, and ultimately the resectability in PDAC. In general, inter-observer agreement is known to be low in PDAC imaging and has been reported to be as low as 7.2–30% [10, 11]. This is also true for experienced radiologists, who show only slightly improved interobserver agreement compared to their less experienced counterparts [11]. Given these numbers, our reported results in the 3D VR group show a relatively high interobserver agreement. This is also reflected in the Fleiss κ range of 0.4–0.7 in our cohort, compared to the κ range of 0.282–0.555 reported by Giannone et al. [10] Of note, the interobserver agreement in the standard 2D PACS group was within this previously reported range. Furthermore, median time needed by the participants to reach a conclusion was only 185 s (VR CT) and 116 s (VR MRI), representing a fast assessment. It is important to note that the study participants were free to use as much time to assess the scans as they liked, and interacted significantly shorter with the standard PACS imaging. The shorter viewing time in the 2D PACS viewing group may be attributed to the individuals’ familiarity with standard 2D assessment of scans. Participants are accustomed to this format which could lead to faster assessments. In contrast, 3D VR viewing invites the viewer to study the scans in more detail, using various tools at hand to freely interact with the scan from multiple angles. Increased exposure time could also be a part of the explanation for improved results in the 3D VR group, as participants take more time to study the scans before coming to a decision. To further assess the value of VR enhanced imaging to evaluate resectability of PDAC, a larger randomised prospective trial is needed, and correlation with perioperative as well as long-term clinical outcomes could shed light on the clinical value of this technology. Confirmation of these findings in a larger trial could support the transition of standard 2D PACS viewing to routine use of VR enhancement technology clinical practice.

As previously reported, interobserver agreement increases especially in cases of borderline resectability [20]. One of the main reasons contributing to this is that resectability represents a continuum rather than clear groups and might also be judged differently by individual surgeons. This issue has been recognised and recently a debate to redefine the terms ‘resectable’, ‘borderline resectable’ and ‘locally advanced’ has been proposed [10]. In our study cohort, comparison of interobserver agreement between the different resectability categories would require a larger study. Analysis of the variation between official MDT report and assessment of vascular infiltration and resectability status in VR reveals roughly three categories of errors. In the first category, imaging quality was insufficient or misleading, as in the case with the previous right hemicolectomy and the metal artefact. This scan was left in the final analysis to omit an imaging selection bias. The second category includes scans which allow for varying interpretations, such as the scan with the abutment of the SMA slightly below 180°. This scan was identified by one participant as R, but according to the guidelines and the MDT decision was seen as BR. The third category includes scans where the participant was not able to identify the tumour as such. Recently, sensitivity for CT and MRI to detect PDAC have been reported to be 75% and 70%, respectively [21]. Nonetheless, participants were able to identify PDAC and its resectability status overall rapidly and reliably, even surpassing the numbers reported in the literature. This is also taking into consideration that the participants were not accustomed to VR image viewing and have not had the chance to complete their learning curve when handling the VR system, although its use is largely intuitive.

A further added difficulty of resectability assessment based on imaging is the introduction of neoadjuvant chemotherapy in BR PDAC, as cross-sectional imaging appears to overestimate residual cancer and underestimate resectability [22]. Whether VR technology can improve assessment post-neoadjuvant chemotherapy could be a rewarding question for future studies in this context.

An interesting result from our study was that the pancreas was not identified on VR CT by all participants, which however did not represent an issue in the second study step. This is likely explained by the fact that also relatively inexperienced colleagues participated in the first study step, whereas only experienced surgeons and radiologists as well as surgeons who underwent subspecialist HPB training were included. Therefore, this result might reflect on the experience of reading cross-sectional imaging data rather than represent a weakness of the technology per se. In general, a lack of recognition of the tumour does not necessarily reflect a shortcoming of the technology but might be linked to the viewer’s experience and understanding. However, no correlation in the second study step was found between work experience and number of correct answers. Based on this finding, it can be assumed that clinicans can potentially use technology regardless of their experience. Of note, participants in this study preferred the use of VR CT over MRI, which could be explained by the fact CT imaging is widespread in daily clinical practice and surgeons might feel more confident interpreting these scans. However, the number of study participants is too low to draw a meaningful conclusion in this regard. Overall, however, there is controversy surrounding the value of MRI and CT for the assessment of PDAC, and it appears that MRI has a slight advantage over CT in terms of tumour detection rate, but CT appears to be superior for the assessment of vascular infiltration [23]. In this study, we have not included positron emission tomography (PET), and adding such scans to a future study could potentially increase the value of the VR system as tumours could be more easily identified.

The main limitations of this study are the relatively small number of participants in the second study step. Although VR enhanced CT imaging lead to better understanding of resectability of PDAC, a randomised prospective study, with a larger number of participants that directly compares VR to standard imaging and incorporates intraoperative outcomes, especially in BR cases, could offer further information on the clinical use of this software. A further future use of this technology could lie in patient education with the VR visualisation to demonstrate the imaging data to the patients, allowing for a better understanding of their disease and its treatment. Furthermore, implementing this technology in MDT could potentially yield effects such as enhanced collaboration among team members of different specialties, a more comprehensive analysis of medical imaging data and improved communication in case discussion. It is possible that the definitions of resectability will change in the future, and for this reason the implementation of a novel VR technology should not solely be based on the number of interobserver agreement, but rather also be based on clinical utility and user experience. In the future, connecting VR-enhanced visualisation data with AI algorithms that allow for automated assessment of vascular tumour contact might even revolutionise resectability assessment and preoperative planning [24, 25].

In conclusion, VR-enhanced CT and MRI visualisation is a promising tool to display abdominal anatomy that is easy for clinicians of varying levels of experience to interpret. Furthermore, there is the potential for VR-enhanced CT and MRI to be implemented as a tool to aid in future surgical planning and oncological treatment of PDAC.

### Supplementary Information

Below is the link to the electronic supplementary material.

Supplementary file1 (XLSX 11 kb)

Supplementary file2 (DOCX 50 kb)

## Data Availability

The raw data supporting the conclusions of this article will be made available by the authors, without undue reservation.
