# Supplementary material for: Assessment of resectability of pancreatic cancer using novel immersive high-performance virtual reality rendering of abdominal computed tomography and magnetic resonance imaging
Source: Int J Comput Assist Radiol Surg. 2024 Jan 22;19(9):1677–87. doi: 10.1007/s11548-023-03048-0 (PMC11365822; doi:10.1007/s11548-023-03048-0)
Supplement: Supplementary file 2 — Supplementary file2 (DOCX 50 kb) [file 11548_2023_3048_MOESM2_ESM.docx]

| **Patient Name** | **Procedure/intraoperative findings** | **Follow up** | **Vessels/bile ducts etc. (radiology)** | **category** |
| --- | --- | --- | --- | --- |
| 1. A.A.  (CT) | Pylorus-preserving Duodenopancreatectomy (Longmire-Traverso, 08/2017)  - no peritoneal carcinosis  - tumor palpable (head of pancreas)  - gall bladder removal  - lymphadenectomy  - coarse (?) mass in the region of the pancreatic head, no exceedance of organ -> decision to resect -> resection R0 | - OP: Pylorus-preserving Duodenopancreatectomy (Longmire-Traverso, 08/2017)  - Chemo until 04/2018  - local relapse 07/2020  - Radiotherapy/Chemo 09/2020  - Palliative Chemo stopped due to Ascites 01/2021  - new lesion with response to therapy  - 04/2021 PET-CT no evidence of new tumor mass, no evidence of peritoneal carcinosis | **vessels**: contact to V. mes. sup. (< 90°) and A. mes. sup. (< 90°), ø contact to V. portae  **bile ducts etc**: dilatation of intra- and extrahepatic bile ducts, hydrops of the gall bladder, expanded Ductus Wirsungianus | Borderline resectable |
| 2. B.S. (CT) | total Pancreatectomy with additional Splenectomy, Lymphadenectomy and segmental resection of the mesenterico-portal axis with end-to-end anastomosis of VMS and V. portae, 04/2020  - no evidence of peritoneal carcinosis or metastases  - voluminous tumor mass palpable (region of the pancreatic head)  - gall bladder removal  - venous stagnation in the omentum  - Lymphadenectomy in the Lig. hepatoduodenale  - pancreatic head and large parts of the corpus are hardened and strongly adherent to the surrounding  - no organ-exceeding growth but the mesenterico-portal axis is strongly adherent to the tumor mass -> no “undertunneling” possible  - arterial vessels do not seem adherent to the tumor -> resection  - End-to-End anastomosis VMS and V. portae | - metastases after the operation, peritoneal carcinosis, rising of tumor markers  - palliative Chemotherapy recommended | **vessels**: direct contact to the first branch of the jejunal vessels (< 90° circumference), complete blocked V. lienalis (near the „confluens“ area), high grade constriction of VMS  - no tumor contact distal to the first jejunal branch, no contact to Truncus coeliacus/A. hepatica communis, broad contact to A.  gastroduodenalis, no contact to A. lienalis and A. mes. sup.  **Bile ducts etc**: expanded Ductus Wirsungianus | Borderline resectable |
| 3. B.J. (CT) | Total Pancreatectomy, Pylorus-preserving, segmental resection of VMS and Lymphadenectomy 08/2019  - advanced tumor situation but „undertunneling» of the mesenterico-portal axis is possible  - suspected tumor infiltration of the Confluens, Infiltration VMS  - pancreatic head resection with venous segmental resection  - frozen section analysis shows R1 resection  - second resection again shows R1 resection -> total Pancreatectomy | - postop. Cholangiosepsis  - adjuvante Chemotherapy recommended (reduced general condition -> wait, 09/2019)  - follow up appointments were recommended (not further documented in the patient’s documents) | **vessels**: extensive contact to Aorta abdominalis, atypical high confluence of the first jejunal vein together with veins coming from the colon transversum, also fine accessory left branch of the hepatic artery coming out of A. gastrica sinistra (the rest matches standard anatomy)  extensive solid tumor contact from the right side to the V. portae confluence/VMS (ca. 3cm, <180°)  **bile ducts etc**: generally extended, hydrops of the gallbladder, apruption of DHC | Borderline resectable |
| 4. B-F. S. (CT) | Duodenopancreatectomy Whipple 07/2018  - no evidence for peritoneal tumor mass  - gall blader resection  - prox. postpyloric Duodenum shows no infiltration -> resection  - strong adherence of the distal VMS and proximal V. portae  - R0 after frozen section analysis  - second resection of Pylorus and Antrum (Whipple) | - relapse 03/2021, ascites, metastases in the liver, peritoneal carcinosis, blockage of V. lienalis  - patient wishes no active therapy -> hospice | Infiltration of prox. Duodenum  **vessels**: dorsal contact up to V. portae and to V. mes. sup.; no contact to the Truncus coeliacus or AMS, no contact to V. lienalis  **bile ducts etc**: extended Ductus pancreaticus (Wirsung) | Borderline resectable |
| 5. D.V. (CT) | Explorative Laparatomy, Duodenopancreatectomy Whipple with Segmental resection of VMS and Reconstruction, Resection of the ileocolic anastomosis with partial resection of the terminal ileum, Ileotransversostomy 09/2020  - resection gallbladder  - no visible peritoneal carcinosis  - anastomosis region strongly adherent to the pancreatic head (suspected tumorinfiltration)  - Lymphadenectomy  - tumor infiltration, distal VMS  - tumor potentially resectable, arterial structures seem to be tumor-free, ileocolic anastomosis resected, as well as a segment of VMS -> complete resection  - R0 resection | - OP 09/2020  - adjuvant Radio/Chemotherapy 09/2020  -01/2021 suspected pulmonary metastasis, FDG enrichment in the liver (unclear dignity)  - radiotherapy until 04/2021  - 07/2021 soft tissue „plus“ in the pancreatic region DD: local relapse, inflammatory reaction | **vessels**: progressive contact to VMS, possible contact to prox.x AMS, as well as pylorus and Ileotransversostomy  **bile ducts etc**: double duct sign, ecstatic bile ducts, hydrops of the gallbladder | Borderline resectable |
| 6. G.B. (CT) | - neoadjuvant radiochemotherapy  revision-laparatomy, assessment of resectability of the pancreatic-head cancer, cholecystectomy 10/2018  - strong inflammatory reaction of the gallbladder and the pancreas (therefore strong adherences) - venous vessels infiltrated, not possible to detangle the veins from the tumor mass -> stop operation, no resection | - 04/2020 palliative situation with progressive peritoneal carcinosis -> supportive therapy, renouncing of life sustaining therapy  - stable tumor situation, no new metastases, stent in DHC due to tumor thrombosis  - punction of ascites  - worsening of general condition | **vessels**: contact to Aorta (< 180°),  short-range contact V. portae, VMS < 180° (no evidence of infiltration); anatomical variation:  A. gastrica sinistra leaves directly from the Aorta prox. of the coeliac trunc, AMS leaves from directly from the coeliac trunc, no contact to A. hepatica communis and A. lienalis;  A small branch of A. hepatica propria shows short ranged contact to the pancreatic head  **Bile ducts etc**: hydrops of the gallbladder, generalised expansion | Borderline resectable |
| 7. B.P.  (CT) | no OP  diagnosed 2019 | Passed 01/2020  - complex instable palliative situation (peritoneal carcinosis, ascites, aspiration pneumonia, tumorous stenosis at the duodenojejunal transition) therapy stopped due to patient’s will | Contact to Fundus of the stomach, Duodenum, peritoneal carcinosis  **vessels**: surrounds A. lienalis, A. gastrica sinistra | not resectable |
| 8. B.M.  (CT) | no OP (Stent for bile ducts)  diagnosed 09/2017 | passed 10/2017  - Adeno-Ca with metastases | Tumor grows into the Omentum minus (organ-exceeding)  **vessels**: tumor alongside A. gastrica sinistra, coeliac trunc extensively in contact, stenosis of A. and V. lienalis, constriction of V. portae  **bile ducts etc**: expanded inside the liver, DHC dilated/sudden termination, expansion of the gallbladder | not resectable |
| 9. B-A. A.  (CT) | no OP  diagnosed 09/2017 | - from 09/2017 palliative Chemotherapy (progressive tumor)  - 11/2017 decision for best-supportive care  - 12/2017 worsening of the general condition, pain exacerbation | (possible metastases)  **vesseks**: dorsal up to coeliac trunc and up to the ventral aortic wall, 360° enclosure of A. hepatica and A. lienalis, enclosure and little obstruction of V. portae and V. mes. sup, near-the-confluence blockage of V. lienalis, little infiltrative mass close to the exit of AMS  **bile ducts etc**: Ductus Wirsungianus expanded | not resectable |
| 10. B.U. (CT) | no OP  diagnosed 03/2017 | - 04/2017 neoadjuvant Chemotherapy, possible progress of tumor  - 07/2017 Progress and peritoneal carcinosis, ascites  - 08/2017 palliative Chemo  - from 09/2017 best supportive care | **Gefässe**: extensive contact of tumor mass to A. hepatica communis (ca. 180°), also to the Duodenum,  Anatomic variation: A. hepatica dextra leaves from AMS, A. hepatica sinistra leaves from the coeliac trunc  **Bile ducts etc**: stagnation inside Ductus pancreaticus, expansion of the intra- and extrahepatic bile ducts | not resectable |
| 11. B.R. (CT) | no OP  diagnosed 11/2016 | - 06/2017 Radiochemo, best supportive care  - 09/2017 hospice | **vessels**: blockage V. portae with following cavernous transformation, circumferential soft tissue mass around the A. mes. sup., from caudal scarce contact to coeliac trunc, compression of V. lienalis, infiltrations of pylorus, suspected lymph node metastases, peritoneal carcinosis possible  **bile ducts etc**: max. dilated ducts, hydrops of the gallblader, Ductus Wirsungianus expanded (little) | not resectable |
| 12. D.M.M. (CT) | no OP  diagnosis 05/2020 | - 05/2020 palliative Chemo, Radiotherapy, add. Tumor mass inside lung (no connection to pancreatic Ca)  - 06/2021 progress tumor with liver metastasis, best supportive care  - still alive, follow up planned in August 2021 | **vessels**: alongside main branch of AMS, 270° contact to VMS (slight constriction over a short range) | not resectable |
| 13. B.H. (CT) | Complex adhesiolysis, pylorus-preserving duodenopancreatectomy Longmire transverso with compartiment II Lymphadenectomy, Cholecystectomy 04/2017  - initial adhesiolysis of malignant tangles (?)  - tumor is detachable from vessels  - R0 in frozen section analysis | - 10/2020 upper gastrointestinal bleeding (ulcers) -> gastroscopy and further care  - no further steps concerning operation and follow up in the patient’s document | **vessels**: high confluence of the first jejunal vein, no infiltration described  **bile ducts etc**: dilated | resectable |
| 14. G.M. (CT) | pylorus-preserving duodenopancreatectomy Longmire transverso 12/2017  - other than known tumor no further pathology, tumor safely resectable, R0 resection | - adjuvant Chemotherapy until 10/2018  - no further information concerning her pancreatic cancer (03/2019) | No evidence of metastases  **vessels**: dilated confluence  **bile ducts etc**: dilated | resectable |
| 15. H-S.U. (CT) | pylorus-preserving duodenopancreatectomy Longmire transverso 03/2021  - no evidence of peritoneal carcinosis, no organ exceeding growth -> resection, R0 | - 05/2021 adjuvant Chemotherapy  - good general condition, no metastases or changes in known liver lesions | Gefässe: no contact to coeliac trunc and AMS, first jejunal branch is free, marginal contact (<90°) to most prox. Part of VMS  **Bile ducts etc**: clear dilatation of bile ducts, hydrops of the gallbladder | resectable |
| 16. N.K. (CT) | duodenopancreatectomy Whipple en bloc with part of the VMS, Kompartiment II Lymphadenectomy, Cholecystectomie 04/2021  - resection VMS -> R0  - no additional -ectomy of the pancreatic rest due to no change in prognosis and worse QoL | - 05/2021 postop. Intraabdominal infection of the wound -> complete healing  - recommendation for additive Chemo, followed by Radio-Chemo  - 2021: in rehabilitation | Contact to duodenum  **vessels**: focal contact to VMS caudal to the first jejunal branch (< 90°), no contact to arterial vessels  **bile ducts etc**: double duct sign with extensive cholestasis, hydrops of the gallbladder, extended bile ducts | resectable |
| 17. H-R.M-J. (CT) | pylorus-preserving duodenopancreatectomy Longmire transverso, Kompartiment II Lymphadenectomy, Cholecystectomie 05/2018  - no peritoneal carcinosis  - R0 resection | passed 06/2018  - end of life situation, complex postop. process  - revisionlapartomy, rest-pancreatectomy  - acute anuric renal failure after hemorrhagic shock (endoluminal bleeding in jejunum), pneumonia | Extensive contact to duodenum  **vessels**: no evidence for tumor infiltration  **bile ducts etc**: intrahepatic massively dilated, DHC extended | resectable |

| **Patientenname** | **OP Befund** | **Verlauf** | **Gefässstatus/Gallenwege etc.** | **Entscheid** |
| --- | --- | --- | --- | --- |
| 1. D.V. (MRI 04/2020) | Explorative Laparatomy, Duodenopancreatectomy Whipple with Segmental resection of VMS and Reconstruction, Resection of the ileocolic anastomosis with partial resection of the terminal ileum, Ileotransversostomy 09/2020  - resection gallbladder  - no visible peritoneal carcinosis  - anastomosis region strongly adherent to the pancreatic head (suspected tumorinfiltration)  - Lymphadenectomy  - tumor infiltration, distal VMS  - tumor potentially resectable, arterial structures seem to be tumor-free, ileocolic anastomosis resected, as well as a segment of VMS -> complete resection  - R0 resection | - OP 09/2020  - adjuvant Radio/Chemotherapy 09/2020  -01/2021 suspected pulmonary metastasis, FDG enrichment in the liver (unclear dignity)  - radiotherapy until 04/2021  - 07/2021 soft tissue „plus“ in the pancreatic region DD: local relapse, inflammatory reaction | Contact to pylorus and duodenum  **vessels**: contact of 180° to proximal VMS with little vessel irregularity, contact to first jejunal branch, 180° contact to AMS  **bile ducts etc**: hydrops gallbladder and extensive dilatation of bile ducts, double duct sign | Borderline resectable (comment: radiology says not resectable) |
| 2. E-G.I. (MR) | Pylorus-preserving partial duodenopancreatectomy Longmire transverso 11/2018  - cholecystectomy  - no organ-exceeding growth -> resection, R0 | - to 05/2019 adjuvant Chemotherapy, stop therapy  - 10/2019 tumor relapse with new suspicious liver lesions  - patient rejects Chemo  - 03/2020 hospice, palliative Chemo still rejected, pain therapy | **vessels**: short-ranged contact to VMS (right-sided venous branch);  anatomical variation: early branching of VMS under the confluence  **bile ducts etc**: hydrops, DHC and Ductus Wirsungianus dilated | Borderline resectable |
| 3. E.M. (MR) | duodenopancreatectomy Longmire transverso 12/2017  - no peritoneal carcinosis  - Cholecystektomy  - no infiltration of the vessels  - in-between V. portae and exit of the right accessory liver artery there is induration palpable (suspected tumor)  - resection R0 | - 07/2018 adjuvant chemotherapy  - 05/2019 metastases  - 06/2020 lung affection (possibly metastases)  - 12/2020 Progress of tumor  - 02/2021 palliative Chemotherapy  - stable CT findings (also concerning the lung lesions), stable soft tissue “plus” around A. hepatica communis  - no further chemotherapies planned  - 04/2021 Dilatation intrahep. bile ducts  - 05/2021 stent | **vesseks**: 90° contact to VMS;  **bile ducts etc**: DHC extended, intrahep. bile ducts extended, | Borderline resectable |
| 4. G.B. (MR) | - neoadjuvant radiochemotherapy  revision-laparatomy, assessment of resectability of the pancreatic-head cancer, cholecystectomy 10/2018  - strong inflammatory reaction of the gallbladder and the pancreas (therefore strong adherences) - venous vessels infiltrated, not possible to detangle the veins from the tumor mass -> stop operation, no resection | - 04/2020 palliative situation with progressive peritoneal carcinosis -> supportive therapy, renouncing of life sustaining therapy  - stable tumor situation, no new metastases, stent in DHC due to tumor thrombosis  - punction of ascites  - worsening of general condition | **vessels**: contact to aorta, to VMS (on the height of the first jejunal branch)  **bile ducts etc**: general dilatation, DHC stops in pancreatic head, hydrops gallbladder, Ductus Wirsungianus extended | Borderline resectable |
| 5. H.R. (MR) | explorative Laparatomy, open Cholecystectomy, Lymphadenectomy and exploration pancreatic head -> stop operation due to locally not resectable tumor (broad tumor-contact to AMS) 11/2018 | - 12/2018 neoadjuvant Radiotherapy  - 02/2019 Gastroenterostomy, palliative Chemo evaluated, partial resection of stomach  - since 2019 no further therapies  - 04/2020 instable palliative situation | **vessels**: solid tumor contact to AMS (< 180°), no evidence of contour-irregularity  **bile ducts etc**: generalised extension, stop DHC in the pancreatic head, distended gallbladder | Borderline resectable |
| 6. B.W. (MR) | no OP  diagnosed 11/2017 | - 12/2017 palliative Chemotherapy  - 03/2018 little regression of pancreatic corpus tumor, persistant contact to vessels  - 06/2018 palliative radiochemotherapy  - until 01/2019 stable to regressive disease  - 03/2019 liver metastasis  - Stenting DHC  - 06/2019 no cholestasis, no delimitable liver metastases, stationary pancreatic ca, obstruction left ureter (may be result of occult peritoneal carcinosis)  - stable disease (since 2019 no further documents) | No evidence of metastases  **vessels**: broad contact to A. lienalis and 180° contact to AMS, 360° enclosure and blockage of the confluence-near VMS , blockage of the venous confluence and V. lienalis  **bile ducts etc**: Ductus Wirsungianus dilated | not resectable |
| 7. B-Z.D. (MR) | no OP  diagnosed 06/2019 | - 12/2019 start palliative Chemotherapy  - 01/2020 pending pathology report to taken liver biopsie (no further information in patient’s documents) | Suspected liver metastases  **vessels**: circular infiltration and stenosis of AMS and A. lienalis, infiltrations up to the coeliac trunc and A. hepatica communis, V. lienalis infiltrated and blocked, stenosis of VMS and little contact to stomach | not resectable |
| 8. D.M.M. (MR) | no OP  diagnosis 05/2020 | - 05/2020 palliative Chemo, Radiotherapy, add. Tumor mass inside lung (no connection to pancreatic Ca)  - 06/2021 progress tumor with liver metastasis, best supportive care  - still alive, follow up planned in August 2021 | Broad contact to Duodenum (Pars horizontalis)  **vessels**: coeliac trunc free from tumor, AMS completely enclosed, contact to VMS 270° with vessel deformity, first jejunal branch completely enclosed (complete obliteration) | not resectable |
| 9. H.E. (MR) | no OP  diagnosed 05/2016 | - until 11/2017 palliative tumor therapy  - 12/2017 complications -> Ileus/Subileus  - 02/2018 tumor progress, best supportive care  - 03/2018 complex palliative situation | **vessels**: confluence is infiltrated and stenosed, contact to coeliac trunc with long-ranged contact to A. lienalis and A. hepatica communis (ca. 180°)  **bile ducts etc**: dilated Ductus pancreaticus | Not resectable |
| 10. J-F.D. (MR) | no OP  diagnosed 01/2016 | passed 07/2017  - 01/2017 primary Chemo with partial remission  - 04/2017 progressing infiltration/stenosis of prox. Duodenum | **vessels**: long-ranged contact to AMS and coeliac trunc, contact to V. portae with prox. stenosis, long-ranged contact to A. hepatica communis | not resectable |
| 11. B-S.E. (MR) | Pylorus-preserving duodenopancreatectomy Longmire transverso 07/2018  - no peritoneal carcinosis  - cholecystectomy  - R0 resection, reconstruction | - 12/2019 local relapse  - 01/2020 Radiochemotherapy  - 03/2020 no new metastases  - 10/2020 relapse, best supportive care | **vessels**: uncertain contact to porto-mesenterial vessels  **bile ducts** **etc**: extension of bile ducts, double duct sign | resectable |
| 12. B.H. (MR) | Complex adhesiolysis, pylorus-preserving duodenopancreatectomy Longmire transverso with compartiment II Lymphadenectomy, Cholecystectomy 04/2017  - initial adhesiolysis of malignant tangles (?)  - tumor is detachable from vessels  - R0 in frozen section analysisGesunden | - 10/2020 upper gastrointestinal bleeding (ulcers) -> gastroscopy and further care  - no further steps concerning operation and follow up in the patient’s document | Duodenum-near lesion  **vessels**: no direct contact to arterial and venous vessels  **Gallenwege etc**: Hydrops gallbladder, Dilatation of intra- and extrahepatic bile ducts, high-grade stenosis of the DHC (Pancreatic head), Dilatation Ductus Wirsungianus, double duct sign | resectable |
| 13. F.V. (MR) | Pylorus-preserving duodenopancreatectomy Longmire transverso, compartiment II lymphadenectomie and Cholecystectomy 04/2020  - no peritoneal carcinosis  - R0 resection | Passed 06/2021  - 10/2020 relpase DD node metastasis with Infiltration of the right ureter  - 06/2021 Exitus, end of life therapy | **vessels**: no certain contact visible  **bile ducts etc**: extended gallbladder and bile ducts, double duct sign | resectable |
| 14. G-F.A. (MR) | Pylorus-preserving duodenopancreatectomy Longmire transverso 03/2017  - questionable infiltration of VMS -> frozen section analysis  - dorsal of A. hepatica communis with indured tissue -> frozen section analysis  - 2nd resection 1cm (R1) Nachresektion 1cm  - uncertain situation -> no complete Pancreatectomy, but reconstruction | - 06/2018 lung metastases  - 03/2019 stable tumor under chemotherapy -> progress, more lung metastases, probably in liver region as well  - until 09/2019 palliative Chemo  - 10/2019 Rehabilitation | **No visible tumor mass!** Little cysts in pancreatic head  **Bile ducts etc**: extensive dilatation of Ductus Wirsungianus | resectable |
| 15. G.M. (MR) | pylorus-preserving duodenopancreatectomy Longmire transverso 12/2017  - other than known tumor no further pathology, tumor safely resectable, R0 resection | - adjuvant Chemotherapy until 10/2018  - no further information concerning her pancreatic cancer (03/2019) | No evidence of metastases  **vessels**: dilated confluence  **bile ducts etc**: dilated | resectable |
| 16. H-S.U. (MR) | pylorus-preserving duodenopancreatectomy Longmire transverso 03/2021  - no evidence of peritoneal carcinosis, no organ exceeding growth -> resection, R0 | - 05/2021 adjuvant Chemotherapy  - good general condition, no metastases or changes in known liver lesions | **vessels**: contact to prox. VMS (< 90°)  **bile ducts etc**: dilated Ductus Wirsungianus and other bile ducts Gallenwege, Hydrops gallbladder | resectable |
| 17. J.M. (MR) | Pylorus-preserving duodenopancreatectomy Longmire transverso, compartiment II lymphadenectomy, cholecystectomy 11/2016  - no peritoneal carcinosis, no ascites  - R0 resection, reconstruction | - 12/2016 until 07/2017 adjuvant Chemo  - 11/2017 local relapse, curative therapy  - until 01/2018 radiochemotherapy,  - 06/2018 new thrombotic stenosis of the portalvenous confluence, reactive thickening of the intestinal wall and distinct ascites, no peritoneal carcinosis, tumor progression with lung and liver metastases  - 10/2018 palliative situation | No association to the duct system  **Bile ducts etc**: generally dilated bile ducts, sudden stop DHC | resectable |
